# Female breast cancer incidence and survival in Utah according to religious preference, 1985–1999

**DOI:** 10.1186/1471-2407-5-49

**Published:** 2005-05-18

**Authors:** Ray M Merrill, Jeffrey A Folsom

**Affiliations:** 1 Department of Health Science, College of Health and Human Performance, Brigham Young University, Provo, Utah, USA

## Abstract

**Background:**

Female breast cancer incidence rates in Utah are among the lowest in the U.S. The influence of the Church of Jesus Christ of Latter-day Saint (LDS or Mormon) religion on these rates, as well as on disease-specific survival, will be explored for individuals diagnosed with breast cancer in Utah from 1985 through 1999.

**Methods:**

Population-based records for incident female breast cancer patients were linked with membership records from the LDS Church to determine religious affiliation and, for LDS Church members, level of religiosity. Incidence rates were age-adjusted to the 2000 U.S. standard population using the direct method. Cox proportional hazards model was used to compare survival among religiously active LDS, less religiously active LDS, and non-LDS with simultaneous adjustment for prognostic factors.

**Results:**

Age-adjusted breast cancer incidence rates were consistently lower for LDS than non-LDS in Utah from 1985 through 1999. Rates were lower among LDS compared with non-LDS across the age span. In 1995–99, the age-adjusted incidence rates were 107.6 (95% CI: 103.9 – 111.3) for LDS women and 130.5 (123.2 – 137.9) for non-LDS women. If non-LDS women in Utah had the same breast cancer risk profile as LDS women, an estimated 214 (4.8%) fewer malignant breast cancer cases would have occurred during 1995–99. With religiously active LDS serving as the reference group, the adjusted death hazard ratio for religiously less active LDS was 1.09 (0.94 – 1.27) and for non-LDS was 0.86 (0.75 – 0.98).

**Conclusion:**

In Utah, LDS lifestyle is associated with lower incidence rates of female breast cancer. However, LDS experience poorer survivability from breast cancer than their non-LDS counterparts. Parity and breastfeeding, while protective factors against breast cancer, may contribute to poorer prognosis of female breast cancer in LDS women.

## Background

Breast cancer is the most frequently diagnosed cancer among women in the United States [1]. Of 668,470 expected female cancer cases in 2004, 215,991 (32.3%) were breast cancer [1]. The incidence of female breast cancer is most common in developed countries such as the U.S. and Western Europe [2], and is higher among whites than other racial and ethnic groups [3]. Among the registries in the Surveillance, Epidemiology, and End Results (SEER) Program of the U.S. National Cancer Institute, female malignant breast cancer incidence rates are lowest in Utah [3], despite approximately 85% of the population being white, non-Hispanic [4].

About 70% of the 2.3-million population in Utah is affiliated with the Latter-day Saint (LDS or Mormon) religion [4,5]. Hence, female breast cancer incidence rates in Utah are largely representative of LDS women and their lifestyle behaviors. A study based on Utah data from 1971 through 1985 showed that LDS women had significantly lower age-adjusted breast cancer incidence rates than did non-LDS women [6]. A recent cross-sectional study involving non-Hispanic white females in Utah compared breast cancer risk factor behaviors between LDS and non-LDS [7]. This study focused on many factors previously shown to influence breast cancer, including age at first birth [8,9], number of pregnancies and number of births (parity) [10], lifetime duration of breastfeeding [10,11], and alcohol consumption [12]. LDS women compared with non-LDS women had a slightly older age at first birth, higher average number of pregnancies and children (parity), and more total years of breastfeeding. LDS women also displayed lower levels of alcohol consumption. A cross-sectional study conducted in Utah in the late 1970s likewise found LDS women to have a later age at first pregnancy, more pregnancies, and lower prevalence of alcohol consumption [13]. However, research has also shown that reproductive factors which may decrease the risk of breast cancer development may explain poorer breast cancer survival [14].

The current study updates the findings of Lyon et al. [6] to the years 1985–99 and also examines female breast cancer survival in Utah according to LDS status and level of religiosity for LDS.

## Methods

Utah Cancer Registry (UCR) data were linked to LDS Church membership records to estimate breast cancer survival according to LDS (religiously active and less active) and non-LDS populations in Utah during 1985–99.

### Utah Cancer Registry

The UCR, established in 1966, has continuously participated in the Surveillance, Epidemiology, and End Results (SEER) Program of the National Cancer Institute since 1973. UCR staff members and local cancer registrars identify incident cases of cancer among Utah residents through routine and systematic review of pathology reports, medical records, radiation therapy records, hospital discharge lists, and vital records. Tumor characteristics including histology, grade, and primary site are coded according to the International Classification of Disease for Oncology-Second Edition (ICDO-2) [15]. Breast cancer-specific death is coded according to the International Classification of Diseases, 9^th ^Revision, as 174.0 – 174.9 [16], and 10^th ^Revision, which uses an alphanumeric system and codes them as C50 [17]. Categories of stage of disease at diagnosis are documented in the Summary Staging Guide of the SEER Program of the National Cancer Institute [18]. Registry records also include information regarding treatment, survival, and patient characteristics such as age at diagnosis, gender, race/ethnicity, and place of residence at diagnosis. Such information is ascertained from specific statements in medical records, reports from private pathology laboratories and radiotherapy units, and death certificates. Cancer surveillance in Utah is conducted in accordance with standards instituted by the SEER Program and the North American Association of Central Cancer Registries [19,20].

In order to provide valid estimates of cancer survival, a high percentage of cancer cases must be routinely followed to ascertain both vital status and date of last contact. UCR records are linked four times each year with records of death certificates from the Office of Vital Records and Health Statistics from the Utah Department of Health. Results from these routine linkages identify cancer patients who have died, regardless of their cause of death. Registry staff members work closely with cancer registrars in local hospitals to document, through systematic review of medical records, the date of last contact for those cancer patients not known to be deceased. Records for these patients are also linked annually with administrative databases, including Medicaid reimbursement claims, Utah driver license records, and Utah voter registration lists; these databases are often a source of updated information regarding vital status and/or date of last contact. According to SEER Program standards, follow-up is considered to be current when the Registry documents that a patient has died, or when the Registry documents that a patient was alive within 18 months of the annual anniversary of the date of original cancer diagnosis. By this definition, 97.9% of the patients in the present study were determined to have complete follow-up at the time data were captured for analysis.

### Linkage of cancer data with LDS church records

UCR records were linked to LDS church membership records to determine membership in the LDS church. A person was identified as a member of the LDS church if the membership record included a baptismal date. The linkage process took place under direct supervision of the Church and the UCR and was conducted in the Church's Member and Statistical Records Department. After the records were linked, all personal identifying information was stripped from the database to ensure confidentiality.

Records were linked using the probabilistic linking program LinkPro [21]. The program calculates probabilities to identify whether a pair of records refers to the same person. Ten variables that were common to both sets of records were used to link the records: first, middle, and last names; birth day; birth month; birth year; gender; zip code; vital status; and maiden name. SOUNDEX versions of the names were used in the matching process, while actual names were used in the review process. Records were linked if they matched on at least seven of the ten variables. Ambiguous links and records that matched on six of the ten variables were manually reviewed. There were approximately 120,000 incident cases of cancer identified in the UCR database from 1973 to 1999 and approximately 6.6 million records in the LDS Church database. There were 81,617 (68%) UCR records linked to a Church membership record. Of these linked records, 74,829 (92%) matched on at least seven of the ten variables.

In addition to information about baptism, LDS church membership records include information on whether born in the church and whether endowed in the temple. To receive one's temple endowment requires conscientious effort toward obedience to the lifestyle prescribed by LDS doctrine [22]. This includes adherence to certain health practices such as not smoking or consuming alcohol, abstention from sexual relationships outside of marriage, and being honest in ones dealings with others [23]. A man or woman aged 19 year or older who is considered worthy in a personal interview with a church authority may receive a temple endowment. Generally LDS men and women receive their endowment prior to serving an LDS mission or at the time of a temple marriage. Previous research has considered the temple endowment as an appropriate surrogate marker for religious commitment among adults in the LDS church [24]. Although the data allowed us to identify LDS church membership and religiosity among LDS women, information about religious preference and religiosity was not available among non-LDS women.

### Data

The present study was based on 9,619 female microscopically confirmed, actively followed malignant breast cancer cases (ICDO-2 site code C50.0:C50.9) diagnosed in Utah from 1985 through 1999. There were 5,234 (54.4%) religiously active LDS, 1,369 (14.2%) less religiously active LDS, and 3,015 (31.4%) non-LDS. In addition to LDS membership and level of religiosity, the following variables were considered: age, marital status, summary stage (pathological), histological grade, number of primary cancers in one individual, and treatment. Because of the high percentage of whites in Utah [4], analyses are limited to whites only.

Age was categorized as 0–34, 35–44, 45–54, 55–64, 65–74, and 75 years and older; marital status was categorized into four groups: never married, married/cohabitating, previously married, and unknown; and calendar years were categorized into four groups: 1985–88, 1989–92, 1993–96, and 1997–99. Number of primary cancers in an individual describes the chronology of diagnosis of all primary malignant cancers over the entire lifetime of the person [19]. Localized tumors are confined to breast, regional tumors are spread to contiguous organs or lymph nodes, and distant tumors are spread to remote organs. Histological grade is defined by the SEER Program Coding Manual and by the International Classification of Diseases for Oncology, Second Edition in increasing level of severity as either low grade (well differentiated), medium grade (moderately differentiated), or high grade (poorly differentiated) [15,19]. These categories are approximately equivalent to the more widely used Gleason grading system as Gleason score 2–4 (low), 5–7 (medium), and 8–10 (high) [19]. Radiation therapy was identified if radiation was a first course of cancer-directed therapy. Surgery was classified as conservative surgery [none; no cancer-directed surgery of primary site; partial mastectomy, not otherwise specified (NOS); less than total mastectomy, NOS], mastectomy [total (simple) mastectomy, NOS; modified mastectomy; radical mastectomy, NOS; extended radical mastectomy], or other surgery (mastectomy, NOS; surgery, NOS) [19].

Cases who had died but had unknown date of death, whose cause of death was determined by death certificate or autopsy only, or who represented a second or later primary cancer were excluded from the survival analysis. After these exclusions, there were 8,731 cases available for this portion of the study.

SEER registries were included in the study if they provided data to the National Cancer Institute during all the years from 1985 through 1999. These registries are as follows: San Francisco-Oakland, Connecticut, Metropolitan Detroit, Hawaii, Iowa, New Mexico, Seattle (Puget Sound), Utah, and Metropolitan Atlanta.

### Statistical analyses

Age-adjusted rates were derived using the direct method and standardized to the 2000 U.S. population. Counts used in the rate calculations were microscopically confirmed. LDS and non-LDS breast cancer incidence rates were compared with SEER incidence rates. Ninety-five percent confidence intervals for the incidence rate calculations were based on standard error estimates from the formula SE(rate) ≈ rate/(cancer cases)^1/2 ^[25]. Population attributable-risk percent is used to estimate the impact of having a certain breast cancer risk profile in Utah.

Among LDS, population values were not available by level of religiosity. Hence, rates are presented for LDS and non-LDS. However, level of religiosity among LDS could be estimated among the cases such that the survival analysis considered religiously active LDS, religiously less active LDS, and non-LDS.

Survival time was calculated as the time interval between diagnosis and date of last information. For deceased cases, the date of last information was the date of death. For cases not known to be deceased, the date of last information was the date that the case was last known to be alive. Cases with death from breast cancer (the outcome of interest), and all other cases were censored at the time of last information. We estimated breast cancer-specific survival for cases where breast cancer is a single cancer primary or the first of multiple cancer primaries.

Bivariate comparisons were evaluated using the chi-square test. Survival estimates were calculated by the Cox proportional hazards method with one-month intervals [26,27]. Statistical significance based on Cox proportional hazards was determined using 95% confidence intervals. The appropriateness of the Cox proportional hazards model was assessed graphically. Statistical analyses were derived in Statistical Analysis System (SAS) software, version 9.0 (SAS Institute Inc., Cary, NC, USA, 2003).

## Results

Bivariate analyses of the association between religion and selected variables for female malignant breast cancer patients in Utah, 1985–99 are displayed in Table 1. Significant differences in the distribution of patients among these groups were observed across age categories, marital status, cancer primaries, and histological grade. Religiously active LDS patients tended to be older and were more likely married or previously married. Religiously less active LDS were more likely to have multiple cancer primaries. The high percentage of unknown histological grade at diagnosis makes this variable impossible to evaluate. The median difference in rates between LDS and non-LDS women across the study years was 20.2 per 100,000. The smallest difference in rates was 8.5 in 1992 and the largest difference in rates was 43 in 1991.

**Figure 1:**
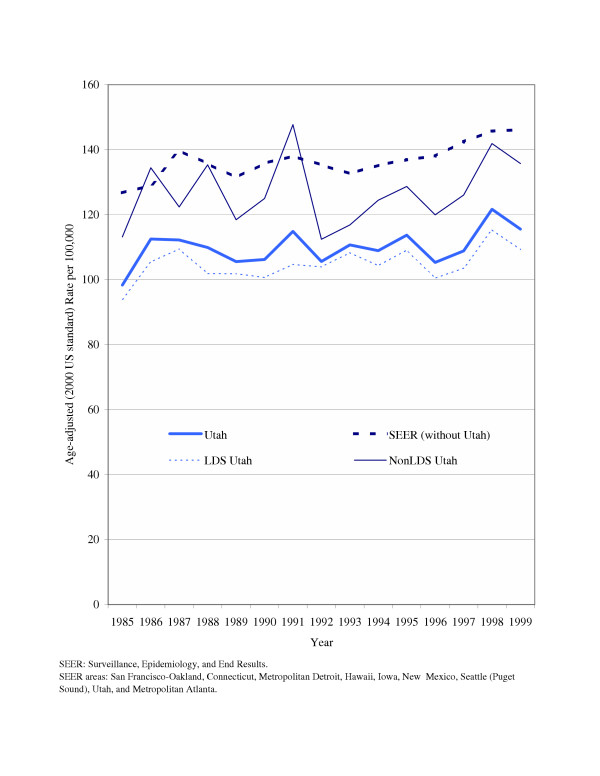
Malignant breast cancer incidence in white women in Utah and SEER (without Utah)

Age-adjusted female malignant breast cancer incidence rates among white women in Utah are presented by LDS status and calendar year in Figure 1. The comparatively low rates in Utah compared with SEER (without Utah) exist across calendar years. The lower rates are primarily explained by LDS women, although non-LDS women also contributed to the lower rates.

**Figure 2:**
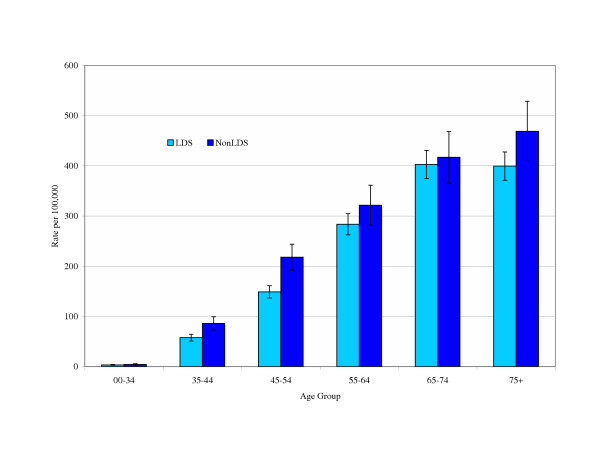
Age-specific malignant breast cancer incidence among white women in Utah according to LDS status, 1995–99

For the combined years 1995 through 1999, lower female malignant breast cancer incidence rates among LDS compared with non-LDS women in Utah were observed across the age span (Figure 2). Age-adjusted breast cancer incidence rates were 107.6 (95% CI: 103.9 – 111.3) for LDS women and 130.5 (123.2 – 137.9) for non-LDS women. In SEER (without Utah) the age-adjusted breast cancer incidence rate was 141.9 (140.9 – 142.9). Hypothetically speaking, if non-LDS women in Utah had the same breast cancer risk profile as LDS women, 214 (4.8%) fewer malignant breast cancer cases would have occurred during 1995–99. If the 9 SEER regions had the same breast cancer risk profile as women in Utah, 12,261 (16.2%) fewer malignant breast cancer cases would have occurred. If the 9 SEER regions had the same breast cancer risk profile as LDS women in Utah, 16,947 (22.4%) fewer malignant breast cancer cases would have occurred.

**Figure 3:**
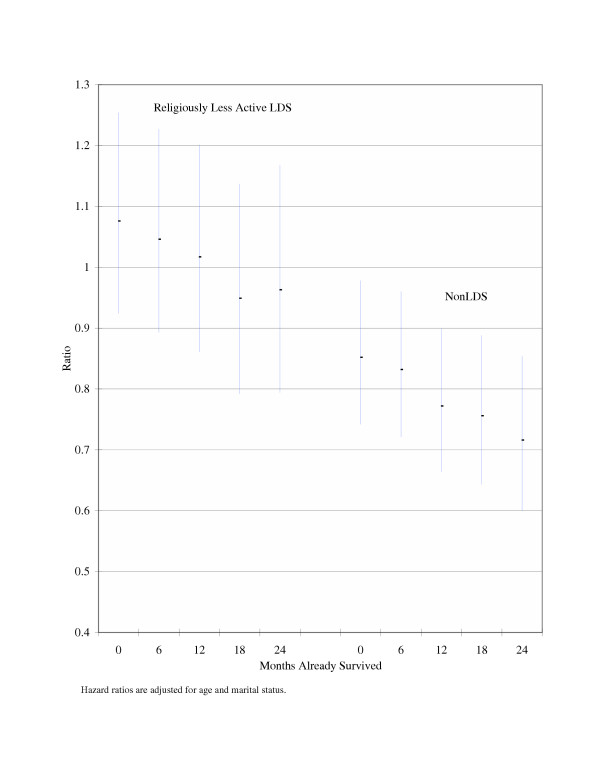
Hazard ratios (95% confidence intervals) of death from breast cancer for religiously less active LDS and non-LDS compared with religiously active LDS conditioned on having already survived *x *months

A proportional hazards model was used to estimate the death hazard for female malignant breast cancer among patients, with religiously active LDS as the referent group. The death hazard ratio for religiously less active LDS was 1.09 (0.94 – 1.27) and for non-LDS was 0.86 (0.75 – 0.98). Adjusted hazard ratios for age and marital status were also computed for patients based on their already having survived 0 months, 6 months, 12 months, 18 months, and 24 months (Figure 3). Religiously less active LDS compared with religiously active LDS showed no significant difference in the death hazards due to breast cancer across each of the conditioned time periods. On the other hand, non-LDS compared with religiously active LDS had significantly lower death hazard rates for breast cancer for each of the conditioned time periods.

## Discussion

Utah presents a unique area for breast cancer studies because of the relatively low incidence of breast cancer among its residents, and the lower risk of breast cancer among members of the LDS church compared with non-LDS in the state. Lifestyle behaviors common among members of the LDS church, such as higher birthrates, higher prevalence and duration of breastfeeding, and lower alcohol consumption have been associated with lower incidence rates of breast cancer. The significantly lower risk of breast cancer among LDS shows the potential preventive effect certain behaviors may have against breast cancer. Yet this study also showed poorer survivability among a population with a traditionally lower incidence rate of breast cancer. It appears that the same factors that are associated with a lower incidence rate of breast cancer are also associated with poorer prognosis once breast cancer is diagnosed. This paradoxical finding is consistent with the results of a study by Korzeniowski and Dyba who reported that "reproductive factors known to decrease the risk of breast cancer development have an adverse effect on prognosis" [14].

Results in the current paper confirm those found by Lyon et al. [6]. Specifically, age-adjusted rates are consistently lower for LDS than non-LDS women. In addition, both LDS and non-LDS women displayed lower breast cancer incidence rates than in SEER. Lower rates among LDS women were observed across the age span. The lower rates among LDS women were likely primarily explained by their having significantly higher average number of pregnancies and children (parity), total years breastfeeding, and lower alcohol consumption [7,13].

Not only did LDS women in Utah experienced lower breast cancer incidence rates than did women in SEER, non-LDS women in Utah also had lower rates. This may be attributed to a conforming effect wherein the non-LDS minority approximates the lifestyle behaviors of the LDS majority to increase their social acceptability [28]. Social and cultural factors influence behaviors such as breastfeeding [29]. Religious preference and the dominant religious preference of one's residence are two independent factors associated with differences in desired number of children [30]. A religious group may have certain reproductive standards, but when they come in contact with a religious group with different standards, their reproductive health attitudes may change [31]. When a majority group exerts moderate social pressure, especially if greater social acceptance is achievable, the minority group is more likely to conform to the majority's values [32]. Each of these factors fits the relationship between LDS and non-LDS in Utah and may explain non-LDS conformity to selected LDS practices.

It should be noted that the linked data did not provide information about religious preference or religiosity among the nearly 30% non-LDS women. However, if the women in the study represent a similar representation as those identified in a recent cross-sectional survey of Utah adult women, approximately 26% of the non-LDS women were religiously active, 41% were less religiously active, and 34% had no religious preference [7]. Daniels et al. showed that the significantly lower average number of pregnancies and children (parity), total years breastfeeding, and higher alcohol consumption in non-LDS women were primarily in less religiously active women and those with no religious preference [7].

The population attributable-risk percent was presented to indicate the amount of breast cancer that could have been avoided under the hypothetical situation that everyone in Utah experienced the same breast cancer risk profile as LDS women. If everyone in SEER had the same breast cancer risk profile as LDS women in Utah, this percentage was 22.4%. In other words, approximately 22.4% of all breast cancer in SEER could have been avoided had women in SEER experienced a similar breast cancer risk profile as LDS women in Utah. Applying this percentage to the U.S. female population in 2004, an estimated 48,382 new cases could have been avoided had women in the U.S. displayed similar breast cancer risk behaviors as LDS women in Utah.

There was not a significant difference in death hazard rates between religiously active and less active LDS. However, there was a significant difference in death hazard rates between religiously active LDS and non-LDS. Lower death hazard rates in non-LDS became more pronounced as we conditioned on months already survived from diagnosis. Two possible explanations may help explain this, proximity to last childbirth and diagnosis during the lactation period.

New studies have indicated that a more recent birth prior to the diagnosis of breast cancer is associated with poorer survivability [30-33]. Older studies had failed to show an association, arguing that poorer survivability was coincidental and likely the result of significant delays in diagnosis [34-37]. Religiously active LDS women generally have more children than their non-LDS counterparts. While a greater number of childbirths is not associated with a worse prognosis in breast cancer [32], this higher parity does raise the likelihood that religiously active LDS women will have a more recent birth than non-LDS at the time of breast cancer diagnosis.

A less conclusive, yet possible contributor to poorer survivability among active LDS, is diagnosis during the lactation period. One study noted that breast cancer diagnosed during the lactation period has been associated with poorer survival among women younger than 45 years [35]. This relationship persisted despite adjustment for nodal status, tumor size, and age. A more recent literature review, however, concluded that no epidemiologic evidence exists to indicate that breastfeeding increases the risk of breast cancer recurring or of a second breast cancer developing [41]. It is biologically plausible that breastfeeding may contribute to a reduction in the development and growth risk of breast cancer. Breastfeeding reduces the number of ovulations proportionally to its intensity, and maintaining a lower estrogen level than that observed during the menstrual cycle [42]; it mobilizes endogenous and exogenous carcinogens present in the ductal and lobular epithelial cell environment [43]; and it reduces pH, levels of estrogens, and local carcinogens of the lobules and ducts [44,45]. Due to the limited and conflicting evidence supporting an association between diagnosis during lactation and poorer prognosis of breast cancer, and in light of biological mechanisms during lactation which may actually limit cancer growth, the possibility should not be ruled out that poorer prognosis associated with diagnosis during lactation may actually be attributable to the childbirth which most lactating women would have recently experienced. Further research is needed to clarify this association.

## Limitations

The temple endowment was used as a surrogate marker for religiosity among LDS women. Although receiving one's temple endowment requires conscientious effort toward obedience to the lifestyle prescribed by LDS doctrine, which includes certain health practices like not smoking or consuming alcohol and abstention from sexual relationships outside of marriage, the data did not allow us to identify whether a previously endowed individual continued to adhere to the doctrine of the church. Yet we assume that given the high level of religious commitment required to receive one's temple endowment, the tendency was for these individuals to continue adherence to the health practices of the LDS religion. A related religiosity measure to the temple endowment was not available for non-LDS women.

We were concerned with the degree of accurate assignment of the cause of death, but the influence of inaccurate specification of cancer death appeared to be very small. Bias may result when considering multiple cancer primaries because religiously active LDS are less likely to smoke cigarettes. Hence, they are less likely to get a cancer related to smoking, where these cancers tend to be more lethal (e.g., lung and pancreatic cancers). There would have been a selection bias if we had considered death from any cancer. However, limiting disease-specific death to breast cancer made the groups more comparable with respect to smoking history.

It was not possible to explore the association between histological grade and survivability because of the large number of patients in the "unknown" category. Nevertheless, the difference in distribution of histological grade among patients with known grade information was very similar and not statistically different (χ^2^(4) = 1.71, p = .789). There is no reason to believe that the "unknown" grade would have been distributed differently among the religious groups.

Finally, specific data on breast cancer risk behaviors was not available on the patient level. However, information was available on whether or not a person had previously received their temple endowment. This measure was used as a surrogate for a host of lifestyle behaviors previously observed among religiously active LDS in Utah, such as higher parity and total time breast feeding and lower prevalence of alcohol consumption.

## Conclusion

LDS lifestyle is associated with lower incidence of breast cancer. The age distribution among LDS breast cancer patients in this study strengthens the argument that higher parity, lifetime breast feeding, and lower alcohol consumption have a preventive effect against breast cancer. Non-LDS in Utah approximate LDS behavior to increase social acceptability, serendipitously contributing to a relatively low breast cancer incidence. LDS showed poorer survival from breast cancer than non-LDS in Utah. Parity and breastfeeding, associated with lower risk of breast cancer, may contribute to poorer prognosis once breast cancer is diagnosed.

## Competing interests

The author(s) declare that they have no competing interests.

## Authors' contributions

Ray M. Merrill conceptualized the study, analyzed the data, and drafted the manuscript. Jeffrey A. Folsom assisted in interpreting the data and writing the manuscript.

## Pre-publication history

The pre-publication history for this paper can be accessed here:



## Figures and Tables

**Table 1:** Bivariate analyses of selected factors among breast cancer patients by LDS status and level of religiosity in Utah, 1985–99

	Religiously Active LDS		Religiously Less Active LDS		Non-LDS		χ^2 ^P value
	No.	%	No.	%	No.	%	
Age at Diagnosis							
00–34	119	2.3	55	4.1	91	3.1	
35–44	463	9.1	165	12.3	465	15.6	
45–54	962	18.8	250	18.6	639	21.5	
55–64	1167	22.9	312	23.2	674	22.6	
65–74	1299	25.5	351	26.1	668	22.4	149.7
75+	1093	21.4	211	15.7	441	14.8	<.0001
Marital Status at Diagnosis							
Never Married	123	2.3	110	8.0	217	7.2	
Married (cohabitating)	3044	58.2	626	45.7	1944	64.4	
Previously Married	2067	39.5	633	46.2	810	26.9	414.9
Unknown	0	0.0	0	0.0	45	1.5	<.0001
Breast Cancer Primaries							
Single	4742	90.6	1200	87.7	2735	90.7	11.8
First of Multiple	492	9.4	169	12.3	281	9.3	.0028
Summary Stage (Pathological)							
Localized	3208	61.3	839	61.3	1859	61.6	
Regional	1756	33.5	443	32.4	982	32.6	
Distant	166	3.2	54	3.9	106	3.5	4.3
Unknown	104	2.0	33	2.4	69	2.3	.6340
Histological Grade							
Low (well-differentiated)	576	11.0	152	11.1	336	11.1	
Medium (moderately diff.)	1796	34.3	463	33.8	1076	35.7	
High (poorly diff./undiff.)	1272	24.3	357	26.1	796	26.4	13.7
Unknown	1590	30.4	397	29.0	808	26.8	.0337
Radiation Therapy							
No	3504	67.0	896	65.5	1961	65.0	3.5
Yes	1730	33.0	473	34.5	1055	35.0	.1736
Surgery							
Conservative Management	1835	35.1	508	37.1	1144	37.9	
Mastectomy	3368	64.3	856	62.5	1858	61.6	8.4
Other Surgery	31	0.6	5	0.4	14	0.5	.0775
Year of Diagnosis							
1985–88	1139	21.8	306	22.4	693	23.0	
1989–92	1282	24.5	374	27.3	732	24.3	
1993–96	1525	29.1	371	27.1	855	28.3	7.8
1997–99	1288	24.6	318	23.2	736	24.4	.2498
